# Abrupt Change from Ionic to Covalent Bonding in Nickel Halides Accompanied by Ligand Field Inversion

**DOI:** 10.1021/acs.inorgchem.4c01547

**Published:** 2024-06-10

**Authors:** Max Flach, Konstantin Hirsch, Tim Gitzinger, Martin Timm, Mayara da Silva Santos, Olesya S. Ablyasova, Markus Kubin, Bernd von Issendorff, J. Tobias Lau, Vicente Zamudio-Bayer

**Affiliations:** † Abteilung für Hochempfindliche Röntgenspektroskopie, Helmholtz-Zentrum Berlin für Materialien and Energie, Berlin 12489, Germany; ‡ Physikalisches Institut, Albert-Ludwigs-Universität Freiburg, Freiburg 79104, Germany

## Abstract

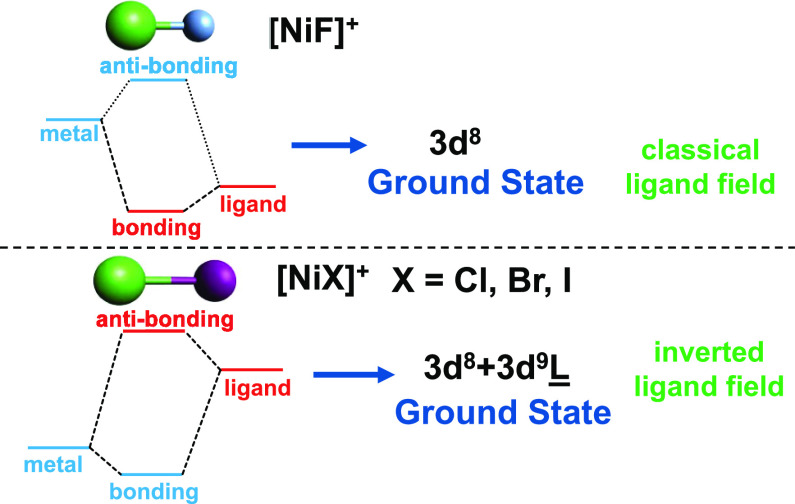

The electronic configuration of transition metal centers and their ligands is crucial for redox reactions in metal catalysis and electrochemistry. We characterize the electronic structure of gas-phase nickel monohalide cations via nickel L_2,3_-edge X-ray absorption spectroscopy. Comparison with multiplet charge-transfer simulations and experimental spectra of selectively prepared nickel monocations in both ground- and excited-state configurations are used to facilitate our analysis. Only for [NiF]^+^ with an assigned ground state of ^3^Π can the bonding be described as predominantly ionic, while the heavier halides with assigned ground states of ^3^Π or ^3^Δ exhibit a predominantly covalent contribution. The increase in covalency is accompanied by a transition from a classical ligand field for [NiF]^+^ to an inverted ligand field for [NiCl]^+^, [NiBr]^+^, and [NiI]^+^, resulting in a leading 3d^9^ L̲ configuration with a ligand hole (L̲) and a 3d occupation indicative of nickel(I) compounds. Hence, the absence of a ligand hole in [NiF]^+^ precludes any ligand-based redox reactions. Additionally, we demonstrate that the shift in energy of the L_3_ resonance is reduced compared to that of isolated atoms upon the formation of covalent compounds.

## Abstract 2

The electronic structure is decisive in determining the redox reactivity of a compound. Here, an abrupt change in the electronic structure of diatomic nickel halide cations from pure 3d^8^ to predominantly 3d^9^ L̲ character, described as nickel(II) and nickel(I), respectively, is observed. While iodine, bromine, and chlorine in these nickel species exhibit a ligand hole and open shell ligands, fluorine in [NiF]^+^ becomes closed-shell, prohibiting ligand-based redox reactions. These distinct differences are induced by ligand field inversion.

## Introduction

Electronic configurations play an important role in the reactivity of late transition metals^1−3^ and therefore define their suitability for their use in electrochemistry as electrode materials used for catalytic processes in renewable energy technologies.^4,5^ While late second- and third-row transition metals are rare, late first-row transition metals are more abundant and are therefore more viable for large-scale applications. Hence, the development of 3d transition metal (e.g., nickel) catalysts to replace catalysts made of 4d and 5d transition metals of the platinum group has gained increasing interest.^6^ Late first-row transition metal halides have been shown to be promising candidates for potent catalytic systems^7−9^ including late transition metals in low oxidation states in the proposed catalytic cycles.^10,11^ Both the electronic configuration of transition metals and the nature of the halogen ligand have been shown to affect the reactivity of transition metal halides.^12−14^ As the need for renewable energy technologies and therefore new electrode materials is high, rational design and optimization may enable a great leap in the development of new effective materials. A detailed understanding of the electronic structure beyond the determination of formal 3d transition metal oxidation states can be of great help in these developments as it can facilitate better predictions and insights into the processes at the center of catalytic activities.^15^ X-ray absorption spectroscopy is a common and widely used element-specific tool to probe the electronic structure in various samples and settings across the spectrum of studies of electrochemical materials and processes. Directly probing the 3d states,^16^ X-ray absorption spectroscopy at transition metal L_3_-edges is often employed to determine oxidation states.^17−20^ Probing the valence occupation of model systems of diatomic transition metal halides and monoatomic transition metals in different electronic configurations can yield unobstructed insights into spectroscopic characteristics that are determined by changes in their electronic structure and may be related to distinct catalytic behavior. Even though the theory of atomic core level spectroscopy is well understood and energy differences upon changes of the 3d occupation have been predicted,^21^ experimental values reported in the literature differ from theory, provided that no empirical fitting parameters are applied. In order to further the development of more complex catalytic materials, we present X-ray spectroscopy data for the most fundamental and simple transition metal halide systems, with the aim of investigating their electronic structure beyond simply identifying their oxidation state. Moreover, the electronic structure of the halide ligand itself is of interest in the context of halogen-atom transfer in nickel photocatalysis.^22,23^

Here, we study diatomic nickel halide cations [NiX]^+^ (X = F, Cl, Br, I) and singly charged nickel cations in their well-defined electronic configurations of 3d^9^ and 3d^8^4s^1^ in the gas phase, accompanied by crystal field and (charge transfer) multiplet simulations.^24−26^ All [NiX]^+^ (X = F, Cl, Br, I) species are in the same charge and formal nickel oxidation state.^27^ However, we find from analyzing the distinctive spectral shapes for 3d^8^4s^1^ and 3d^9^ configurations that the nickel center in nickel halides undergoes a change of the electronic configuration from predominantly 3d^8^ to predominantly 3d^9^L̲ with the change in covalency from [NiF]^+^ to [NiI]^+^ within the same formal oxidation state. We also probed, via nickel L-edge XAS, Ni^+^ monoatomic cations prepared in two electronic states, 3d^8^4s^14^F_9/2_ and 3d^9^^2^D_5/2_. These 3d occupations correspond to pure nickel(II) (3d^8^) and nickel(I) (3d^9^) oxidation states according to the ionic approximation in 3d transition metals, which is the basis for the definition of formal oxidation states.^27^ In that model, integer numbers of electrons are attributed to each constituent. The median excitation energy shift between these two well-defined states of 2.3 eV is in line with the predicted shift by Hartree–Fock-based atomic multiplet theory for exactly one integer change in 3d occupation.^21^

## Methods

The experiments were performed at the Ion Trap endstation,^28,29^ located at beamline UE52-PGM of the synchrotron facility BESSY II operated by Helmholtz-Zentrum Berlin. Both available ion sources at the endstation, magnetron sputtering and electrospray ionization (ESI), were used. The magnetron sputtering ion source was used for the production of nickel cations, Ni^+^, and diatomic nickel argon cations, [NiAr]^+^. The argon attachment was achieved by cooling the aggregation volume to approximately 120 K using a liquid nitrogen cooling jacket. For the formation of the nickel fluoride [NiF]^+^, nickel chloride [NiCl]^+^, and nickel bromide [NiBr]^+^ diatomic cations from the Ni^+^ precursor, gaseous CH_3_F, CH_2_Cl_2_, and CBr_2_F_2_ were introduced into a collision cell downstream from the magnetron source.^30^ Additionally, the ESI source was used to produce nickel bromide [NiBr]^+^ and nickel iodide [NiI]^+^ diatomic cations by spraying commercially available nickel(II) bromide and nickel(II) iodide in ultrapure water, respectively (see Supporting Information). From the comparison of [NiBr]^+^ spectra obtained using the two different sources, no noticeable influence of the source on the spectra was observed, as expected from our previous work.^29^ Hence, the prepared states of the ions are the same for both sources.

After production, the ions are guided by quadrupole and hexapole ion guides and mass-selected by a quadrupole mass filter before being stored for accumulation in a linear radio frequency (Paul) ion trap. For thermalization, cryogenic helium buffer gas is applied to cool the ions in the trap to a temperature of around 20 K.^31,32^

Sputtering the nickel target produced an ion beam of nickel cations with a mixture of the ground-state configuration 3d^9^ and the metastable first excited configuration 3d^8^4s^1^.^33,34^ For the exclusive production of nickel cations in their ground-state configuration, the cold magnetron source was first set to mainly produce [NiAr]^+^ diatomic cations. The collision-induced dissociation of the diatomic molecules into Ni^+^ was then induced in the ion trap by using relatively harsh trapping conditions. Specifically, a trapping potential of −9 V instead of −1.4 V was used, thus increasing the kinetic energy of the [NiAr]^+^ diatomic cations, resulting in dissociation into the ground-state configuration Ni^+^ 3d^9^ and Ar.^35^ To obtain the X-ray absorption spectra of Ni^+^ in the 3d^8^4s^1^ configuration, the Ni^+^ 3d^9^ contribution as deduced from a fit was subtracted from the spectrum obtained with both configurations present in the ion trap (see Supporting Information).

X-ray absorption spectra of atomic nickel cations were simulated using Hartree–Fock-based multiplet calculations,^24^ as implemented in the Missing software package.^26^ Multiplet calculations of the two configurations were Gaussian-broadened by 200 meV to fit the experimental bandwidth and shifted by −3.4 eV to fit the corresponding measured spectra. To simulate the spectra of the cationic nickel halides, charge transfer multiplet simulations were performed using CTM4XAS (version 5.5).^25^ All charge transfer calculations were broadened by 200 meV to fit the experimental spectra and red-shifted by about 1 eV (see Table S1) to match the measured energy position of the absorption maximum, see Supporting Information.

## Results

### Electronic State-Resolved X-ray Absorption Spectra of Ni^+^

**Figure 1:**
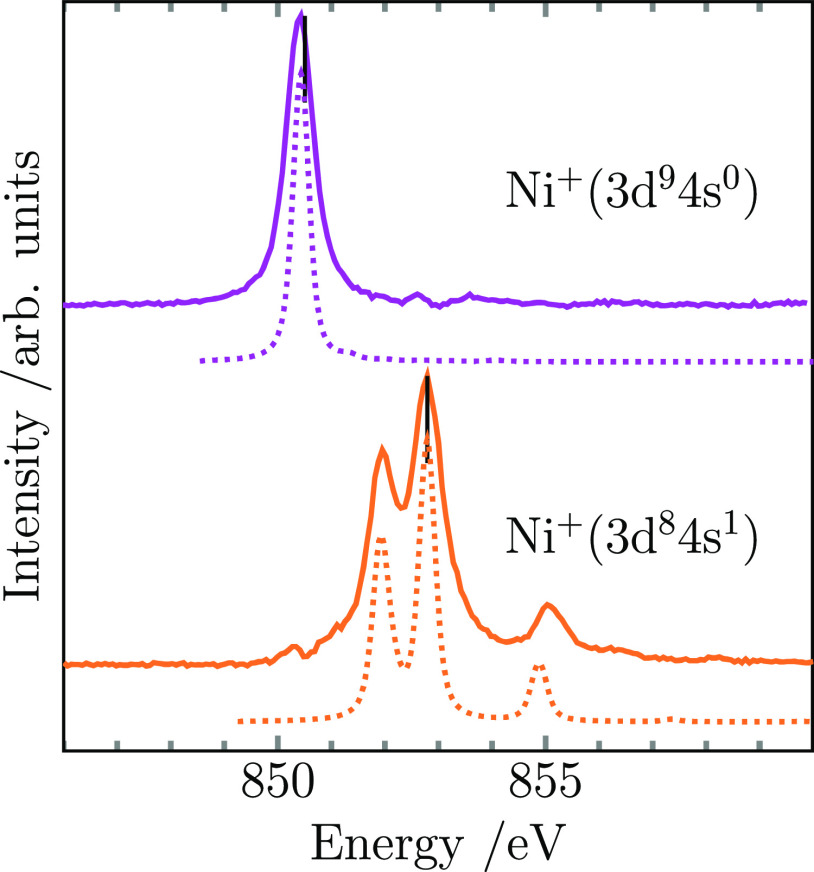
Ion yield spectra at the nickel L_3_-edge of nickel cations (solid lines) in their two lowest-lying electronic configurations. Black vertical markers indicate the median energy of the L_3_ multiplets. Also shown are simulated spectra from multiplet calculations for ground state (Ni^+^ 3d^9^) ^2^D_5/2_ and first excited state (Ni^+^ 3d^8^4s^1^) ^4^F_9/2_ (dotted lines). The simulated spectra have been shifted by −3.4 eV.

Figure 1 shows the measured ion yield spectra at the nickel L_3_-edge for nickel monoatomic cations in two distinct electronic configurations. The spectrum at the top corresponds to Ni^+^ that is formed upon dissociation of [NiAr]^+^ complexes, with the aim of quenching any electronic excitations to the ground state (see Supporting Information). As expected, the spectrum consists of only one absorption line in agreement with the 3d^9^^2^D_5/2_ ground state featuring a single vacancy in its 3d valence shell (timely energy calibration before and after the measurements explains the deviation of the excitation energy position compared to previous work).^36,37^ Furthermore, the spectrum agrees well with the simulated Hartree–Fock spectrum of Ni^+^ with a 3d^9^ initial-state configuration that is additionally shown in the figure for comparison.

Nickel cations are produced in the magnetron source in both ground and first excited electronic-state configurations, namely, 3d^8^4s^1^ and 3d^9^. Consequently, the resulting X-ray absorption spectrum is a superposition of nickel in both electronic configurations. By subtracting the 3d^9^ contribution (see Supporting Information), we can obtain the ion yield spectrum of Ni^+^ in a pure 3d^8^4s^1^ configuration, which is presented at the bottom of Figure 1 alongside the simulated Hartree–Fock spectrum of Ni^+^ with a 3d^8^4s^1^ initial-state electronic configuration. The agreement of the simulation and ion yield spectrum confirms that the remaining contribution is solely due to Ni^+^ in its first excited state 3d^8^4s^14^F_9/2_. The three observed lines originate from the three dipole-allowed transitions to core-excited states with a c 3d^9^4s^1^ configuration, namely, ^4^F_9/2_, and two configurations forming ^4^D_7/2_. The same three main lines were observed in the Ni L_3_-edge absorption spectrum of evaporated nickel atoms in the ^3^F low-lying excited state,^36^ thus demonstrating that not only the energy shift but also the absolute energies of the Ni L_3_ resonance from 3d^9^ to 3d^8^ initial-state d-occupation are independent of the charge state. From the lower-resolution spectra covering the whole Ni L_2,3_-edge range (see Supporting Information) and after normalizing to the yield intensity above the core-ionization threshold, the intensity ratio of the L_3_ resonance between 3d^8^4s^1^ and 3d^9^ configurations is 2:1, as expected due to a doubling of the number of 3d holes.^38−40^ Moreover, the L_3_ resonances of the two configurations are 2.3 ± 0.2 eV apart, as evaluated by the median L_3_ excitation energy.

### Nickel L_3_-Edge XAS of [NiX]^+^ (X = F, Cl, Br, I)

**Figure 2:**
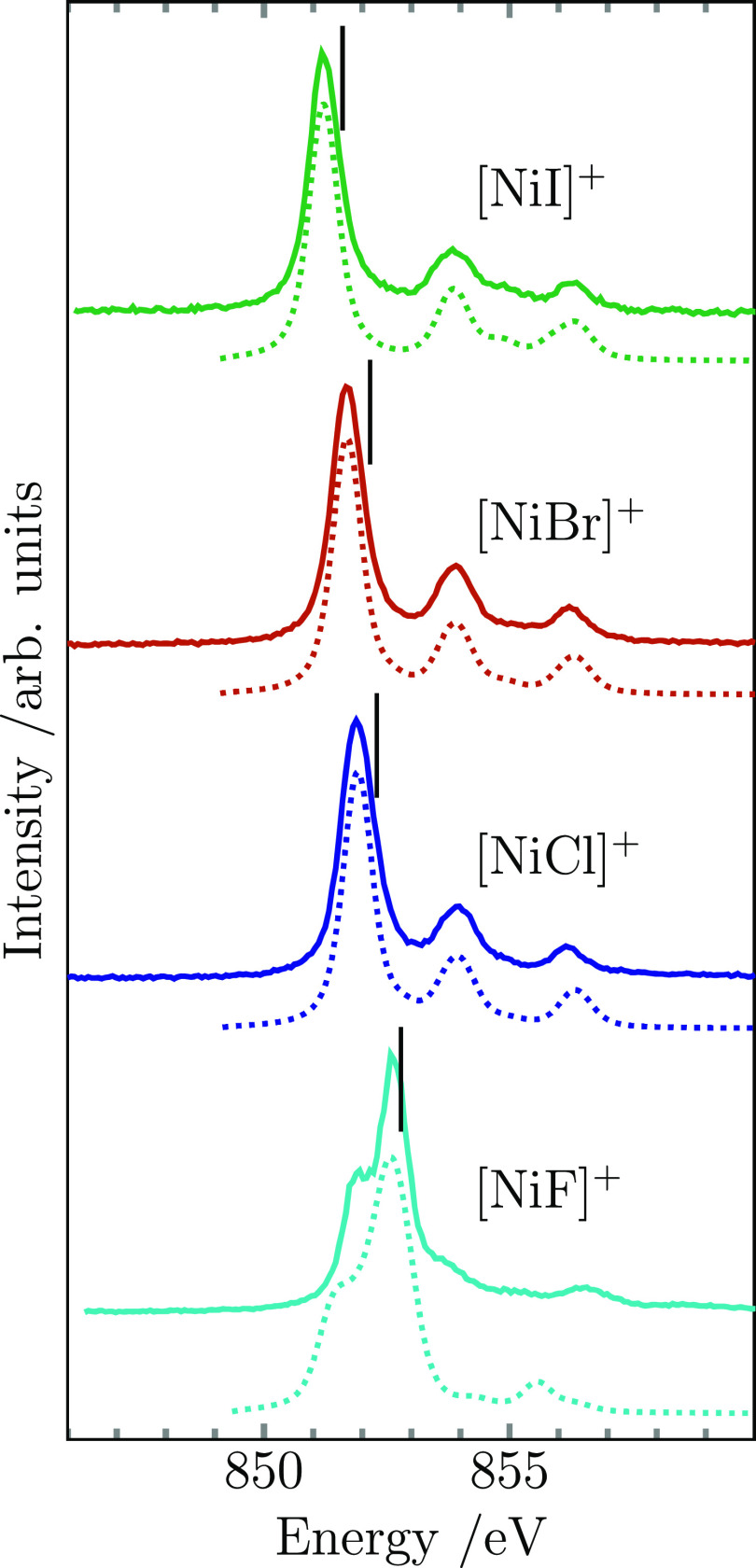
Measured Ni L_3_ ion yield spectra of [NiX]^+^ (X = F, Cl, Br, I) (solid lines) together with their respective simulated spectra obtained from multiplet calculations for [NiF]^+^ and charge transfer multiplet calculations for the rest of the nickel halide series (dotted lines). Black vertical markers indicate the median energy of the measured Ni L_3_ resonance. The simulated spectra have been rigidly shifted in energy to match the measured spectra (see Supporting Information).

The nickel L_3_-edge ion yield spectra of the [NiX]^+^ (X = F, Cl, Br, I) series are shown in Figure 2, together with their best matching charge transfer multiplet simulations (for details, see Supporting Information). It can be seen that the spectrum of [NiF]^+^ is unique within the series, while the number of absorption lines and their relative intensities are essentially identical for the diatomic cations of the other three halides. The spectrum of [NiF]^+^ is dominated by two lines of comparable intensity that are also close in energy. A weak shoulder on the high-energy side of the main line is followed by a very weak satellite at higher energy that is not reproduced well by the simulation. In contrast, the other three nickel halide spectra consist of one single leading strong absorption line, well-separated in energy from two satellite lines of considerably lower intensity. While the energy position of the two satellites remains constant along the series, the main line experiences a red shift from [NiCl]^+^ through [NiI]^+^. This results in a shift of the excitation energy median of the Ni L_3_ resonances toward lower energy, see Table 1. The excitation energy median of [NiCl]^+^ is also red-shifted compared to the one of [NiF]^+^, with the overall shift along the whole series amounting to −1.2 eV between [NiF]^+^ and [NiI]^+^. The respective charge transfer multiplet simulations are in good agreement with the experimental spectra, also reproducing the energy separation of the main line and the satellites well for [NiX]^+^ (X = Cl, Br, I). The relevant parameters are tabulated in Table S1.

**Table 1:** Median L_3_ Energies and Median L_3_ Energy Shifts ΔE(L_3_ Median) Relative to the L_3_ Median Position of Ni^+^(3d^8^4s^1^) for Ni^+^ (3d^9^4s^0^) and the [NiX]^+^ Series.^a^ Additionally Given Are Charge Transfer Energies Δ_CT_ for the Best-Matching CTM Simulations.

system	L_3_ median/eV	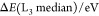	Δ_CT_/eV
Ni^+^ (3d^8^4s^1^)	852.8	0	
[NiF]^+^	852.8	0	
[NiCl]^+^	852.3	–0.5	–0.6
[NiBr]^+^	852.2	–0.6	–0.7
[NiI]^+^	851.6	–1.2	–0.75
Ni^+^ (3d^9^4s^0^)	850.5	–2.3	

a The uncertainty of the L_3_ median energy is ±0.2 eV.

## Discussion

The excitation energy of the metal L_2,3_-edges is often used to identify the oxidation states of transition metals. This is because the concept of oxidation state for 3d transition metals is closely related to their 3d orbital occupation,^41,42^ and X-ray spectroscopy at the metal L_2,3_-edges mainly probes this 3d occupation. The two nickel cation species prepared differ in their 3d orbital occupation, which is why they can be regarded as two distinct oxidation states of nickel. The nickel cation with the ground-state 3d^9^ occupation corresponds to nickel(I), whereas the first excited-state 3d^8^ occupation can be considered nickel(II). The difference in excitation energy between the 3d^8^, i.e., nickel(II), and 3d^9^, i.e., nickel(I), initial-state 3d occupation is independent of the charge state as excitation energies agree with those reported for neutral nickel atoms.^36^ As expected, the excitation energy increases with an increasing oxidation state. The observed shift in the Ni L_3_ energy of 2.3 ± 0.2 eV from nickel(I) to nickel(II) is at least twice as large as the typical shift of 0.9 eV reported in the literature for various nickel compounds.^43−46^ This clear difference is a result of the nephelauxetic effect,^47^ where bond formation leads to 3d electron cloud expansion and thus a reduction of the 2p core-hole to 3d valence Coulomb interaction *U*_pd_, which is the dominant contribution to the energy shift of the L_3_ resonance.^21^ Additionally, it is well-established that in transition metal compounds, an oxidation state can cover a range less than unity of fractional occupations of the respective 3d orbitals.^40,48^ This, in turn, results in reduced shifts of the L_2,3_ edge.^29^ It should also be noted that the L_3_-edge excitation energy of [NiX]^+^ (X = Cl, Br, I) is lower than that for reported nickel(I) in literature,^43^ see Table 1.

**Figure 3:**
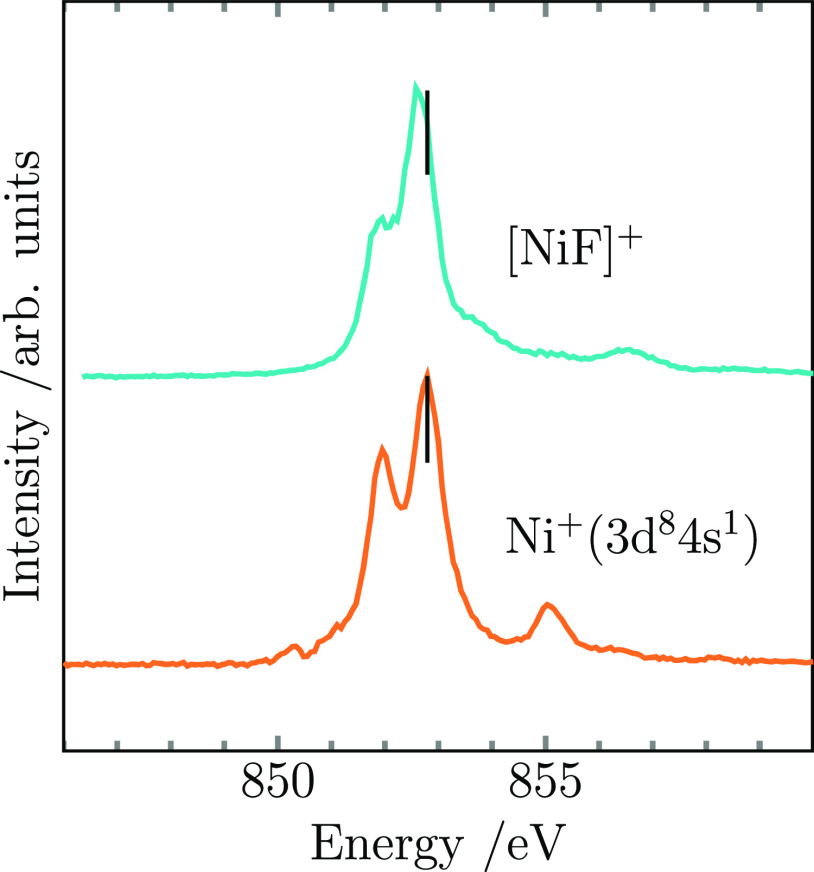
Ion yield spectra at the nickel L_3_-edge of the monoatomic Ni^+^ in the electronic configuration of 3d^8^4s^14^F_9/2_ together with the spectrum of the diatomic [NiF]^+^ (solid lines). The excitation energy median position (black vertical markers) of both spectra coincides, indicating a dominant 3d^8^ character in [NiF]^+^ as well.

Consistent with the same formal oxidation state, the Ni L_3_ excitation energy of [Ni^II^F]^+^ of 852.8 eV was found to be identical to that of the nickel cation with a 3d^8^ occupation corresponding to nickel(II). Furthermore, there is also a strong similarity in spectral shape between both species, see Figure 3. Accordingly, the best agreement of measured and simulated spectral shape at the Ni L_3_ resonance for [NiF]^+^ was achieved for a multiplet simulation with a pure nickel 3d^8^ occupation in a crystal field of *C*_∞*v*_ symmetry. We can therefore establish that charge transfer in [Ni^II^F]^+^ plays no considerable role. The crystal-field splitting corresponds to a (3dδ)^4^ (3dπ*)^3^ (3dσ*)^1^ molecular configuration in agreement with the ^3^Π ground state predicted in the literature^49^ (see Supporting Information for details). In summary, fluorine in [NiF]^+^ behaves like a textbook innocent ligand with a formal oxidation state of −1. This is consistent with the conclusions reached by the theoretical comparison of oxo and fluoro ligands.^50^

**Figure 4:**
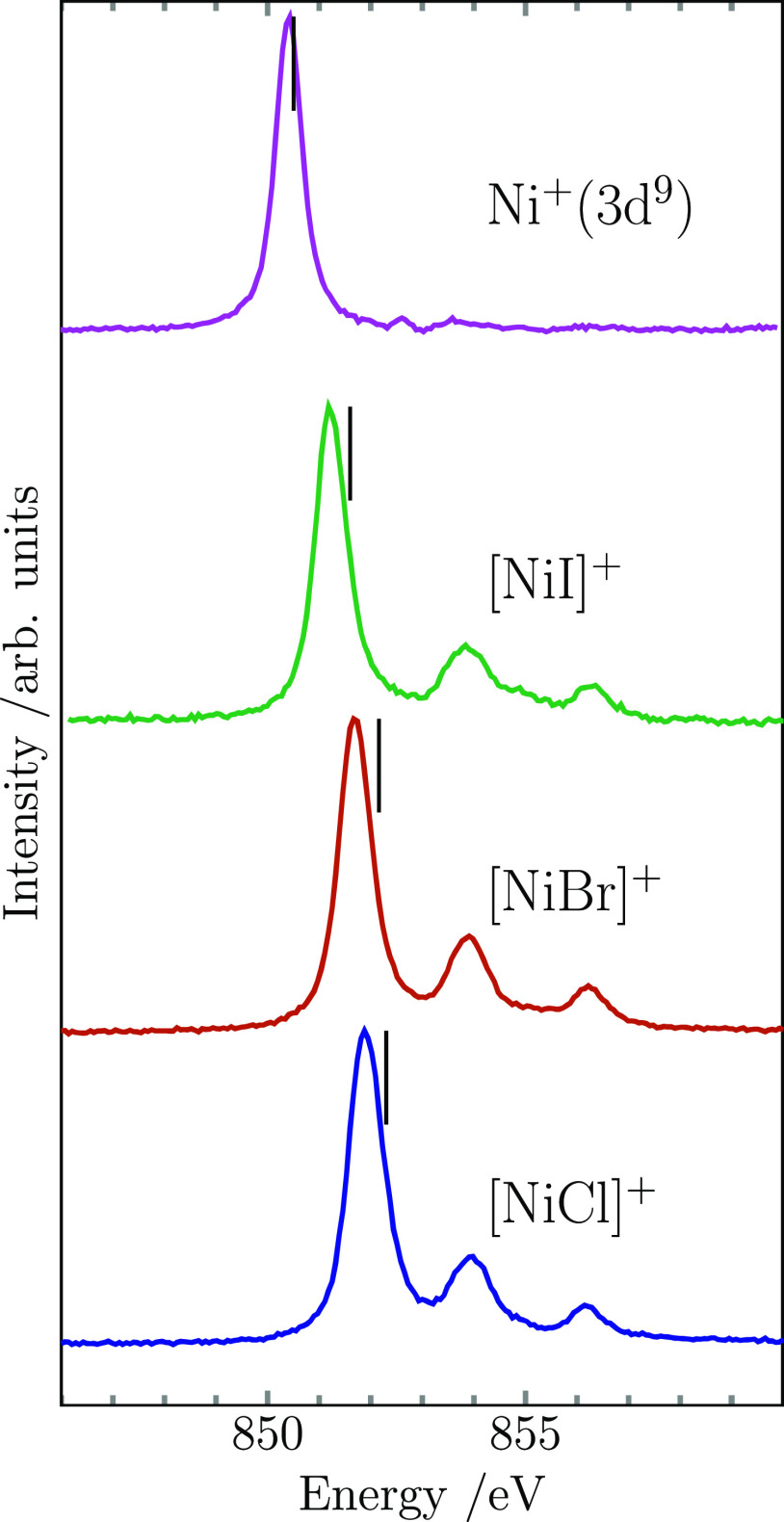
Ion yield spectra at the nickel L_3_-edge of the monoatomic Ni^+^ in the electronic configuration of 3d^9^^2^D_5/2_ together with the spectra of diatomic halides [NiX]^+^ (X = Cl, Br, I) showing a dominant 3d^9^L̲ character (solid lines). The median of the excitation energy (black vertical markers) shifts from [NiCl]^+^ through Ni^+^ to lower energies.

Moving along the halide series to [NiCl]^+^, there is a red shift of the L_3_ excitation energy and a very pronounced change in the spectral shape. While the shift in excitation energy increases along the series, first to [NiBr]^+^ and then to [NiI]^+^, the spectral shape of the nickel L_3_ resonance remains essentially unchanged. The spectral shape appearing for [NiCl]^+^, [NiBr]^+^, and [NiI]^+^ was successfully reproduced in the simulated spectra by choosing a negative charge-transfer energy Δ_CT_, which leads to a multiconfigurational character with the two configurations 3d^9^L̲ and 3d^8^ contributing to the ground-state wave function. The leading d^9^L̲ contribution, which is clearly lower in energy than the 3d^8^ contribution as quantified by charge-transfer energy Δ_CT_, results in a single main line similar to the nickel cation with a 3d^9^ configuration, however with additional satellites higher in energy, see Figure 4. Most remarkably is that the 3d^9^L̲ configuration, which includes an electron vacancy or hole at the ligand, lies considerably lower in energy than the 3d^8^ configuration in [NiI]^+^ through [NiCl]^+^ (see Δ_CT_ in Table S1). The exceptional electronic structure of [NiF]^+^ in the halide series, with only the 3d^8^ configuration contributing to the ground-state wave function, is also observed in bulk nickel halides, with NiF_2_ being an intermediate between a Mott–Hubbard insulator and a charge-transfer semiconductor, while the other nickel dihalides are all pure charge-transfer semiconductors.^51^ The observed trends along the halide series are consistent with the reported 3d hole concentration decrease and accompanying covalency increase in bulk nickel dihalides along the halide series probed via nickel 2p X-ray photoelectron spectroscopy.^52,53^

**Figure 5:**
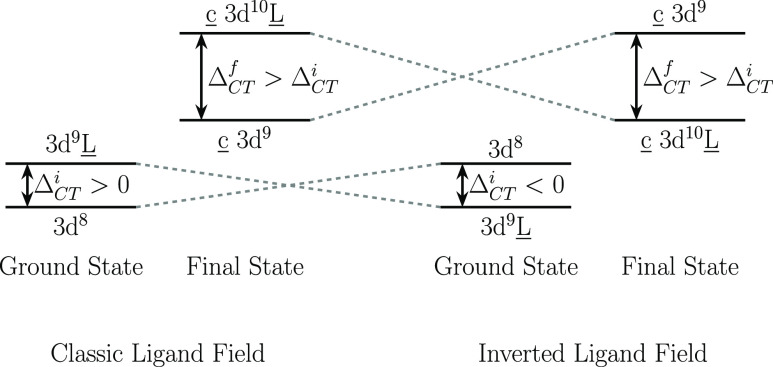
Schematic of the electronic configuration order in the charge-transfer multiplet model for a classic (left) and an inverted ligand field (right). The energy separation between electronic configurations with and without a ligand hole is denoted by Δ_CT_^i^ and Δ_CT_^f^ for the initial and core-excited final states, respectively.

A thorough examination of the nickel L_3_ resonance of nickel chloride, bromide, and iodide reveals that the only considerable change is a red shift of the main absorption band, while the energy position of the satellites remains constant. This change results in a monotonous increase of the energy separation between the main line and the satellites when varying the halide ligand from chlorine through iodine. This trend was successfully reproduced in the simulated spectra and results from a lowering in energy of the 3d^9^L̲ configuration with respect to the 3d^8^ configuration in the core-hole excited state. In other words, a final state with an electron vacancy at the halide ligand becomes increasingly energetically favorable as its electronegativity decreases, see Figure 5.

In the charge-transfer multiplet model, satellites appear in the spectrum when an additional electronic configuration is needed to describe the electronic state of the system.^54^ In the case of a classic ligand field, the additional electronic configuration includes a ligand vacancy and lies higher in energy by charge transfer Δ_CT_ > 0 (see Figure 5). In an inverted ligand-field situation, however, the configuration with the ligand hole lies lower in energy due to a negative charge transfer Δ_CT_. Therefore, in the spectra of inverted ligand-field [NiX]^+^ (X = Cl, Br, I), the observed satellites result from the higher-lying 3d^8^ configuration analogue to the ones previously observed in isovalent negative charge-transfer copper compounds.^54,55^ Their shape is not identical since in copper compounds the shape of the satellites results from multiplets of the core-hole excited c 3d^9^ configuration, while in the nickel halide cations, the shape is determined by the splitting of the 3d orbitals in the linear symmetry of the diatomic molecules into δ, π, and σ components. Consequently, three different hopping parameters were required for good agreement between measured and simulated spectra (see 1 in Supporting Information). Since in *C*_∞*v*_ symmetry the ligand 2p orbitals cannot mix with the 3dδ, the hopping parameter for 3dδ is zero, and only two satellites are observed. When orbital mixing involving 3dδ is symmetry-allowed, a richer satellite structure has been observed.^56^ The absolute energies of the satellites remain essentially constant from chloride to bromide to iodide because, in the case of the 3d^8^ configuration, only 3d orbitals, which are localized at the nickel site, are involved. In contrast, the 3d^9^L̲ configuration involves orbitals from the ligand and might explain its energy dependence with respect to the nature of the ligand. For these heavier halides, the splitting of the δ, π*, and σ* molecular orbitals is strongly reduced, but the sequence remains unchanged, which indicates ^3^Π states with low-lying ^3^Δ states, comparable to the situation observed in the neutral species^57−59^ (see Supporting Information for details).

The comparison with the previously investigated iron halide series shows a similar correlation at both the L-edge^29^ and K-edge^15,60^ of excitation energy and covalency. Furthermore, within their respective halide series, the fluorides show the weakest perturbation of the transition metal 3d orbitals compared with that of the isolated metal atoms. However, while in iron fluoride some charge transfer was required to describe the ground state, in the case of nickel fluoride, one dominant configuration without charge transfer shows the best agreement. Hence, the unique electronic structure of [NiF]^+^ stands out within the iron and nickel halide series. This is consistent with the known tendency of fluorine acting as a π donor to form weaker bonds with later transition metals.^7^ Overall, an increasing role of a second 3d occupation due to charge transfer from fluoride to iodide was observed for both iron and nickel. But while in iron this happens in a gradual manner along the halide series, for nickel, there is an abrupt change between the fluoride and the other halides, as described above.

Considering that the dominant configuration of the nickel halides with chlorine, bromine, and iodine is 3d^9^L̲, as evidenced by the spectral shape and excitation energy of the Ni L_3_ resonance, it could be argued that a physical oxidation state nickel(I) is the more appropriate description. This is remarkable since +1 is one of the less common oxidation states of nickel,^61^ and the only recently reported nickel L_3_-edge spectrum of a confirmed nickel(I) compound is also dominated by a leading single resonance.^62−64^ Therefore, in [NiX]^+^ (X = Cl, Br, I), the halogen ligands are noninnocent since their corresponding physical oxidation state is 0 −instead of −1, as expected from electronegativity differences—a further example of the ubiquity of noninnocent behavior of ligands.^65^ This also aligns well with the general tendency of nickel as a late transition metal with relatively deep-lying 3d orbitals to form compounds with an inverted ligand field where the ligand has a greater contribution to the lowest unoccupied electronic states.^40,46,66^ An inverted ligand field in nickel chloride, bromide, and iodide cations but not for fluoride is consistent with the first ionization energies of the former being considerably (≤5 eV) and that of fluorine being only slightly (740 meV) lower than the second ionization energy of nickel.^67^ Since states with a considerable contribution of the ligand to the lowest unoccupied states are the relevant states that participate in chemical reactions, a greater tendency for ligand redox activity can be expected.^68,69^ In contrast to [NiF]^+^, the distinctly inverted ligand field in [NiBr]^+^ and [NiI]^+^ might also play a role in the activation of alkanes by these two ions that transpires mainly via dehydrogenation and leaving the nickel halogen bond intact.^12^ Moreover, it is worth noting that elusive low-valent nickel intermediates have been found to play a major role in nickel-catalyzed cross-coupling reactions.^70^ Hence, the synthesis of compounds with nickel(I) halides, resembling the aforementioned intermediates, is an active research field.^71^ As a final remark, the difference between [NiF]^+^ and [NiI]^+^ without and with a ligand hole, respectively, can be considered in analogy to the case of transition metal oxo and transition metal oxyl compounds. Identifying the active species in the dioxygen formation step in photosystem II, by distinguishing between oxo and oxyl species through spectroscopy, is a major challenge in understanding the complete reaction cycle.^72^

## Conclusions

In this work, we provide spectroscopic evidence that nickel halides change their leading electronic configuration within the same formal oxidation and charge state from exclusively 3d^8^ in [NiF]^+^ to 3d^9^L̲ in [NiX]^+^ (X = Cl, Br, I). All nickel chloride through iodide species exhibit an inverted ligand field, as demonstrated by their negative charge-transfer energy Δ_CT_, cf. Table 1. However, the configuration mixing increases as the charge-transfer energy Δ_CT_ decreases along the series. The increase in 3d occupation from eight to nine is associated with a red shift of the nickel L_3_ resonance by up to −1.2 eV, which is comparable to a previously reported value of about 0.9 eV for changing of the formal oxidation state.^73−76^ Differences between midrow and late transition metals are observed by comparing the nickel halide series in this study with the iron halides in our previous study.^29^ The late transition metal nickel has deeper-lying 3d orbitals, resulting in larger shifts of the L_3_ excitation energy caused by changes in 3d occupation when comparing nickel to iron halides. Furthermore, we quantified the excitation energy shift at the L_3_-edge of the Ni^+^ cation between ground-state configuration 3d^9^ and first excited configuration 3d^8^4s^1^, which, according to their 3d occupation, represent nickel in formal oxidation states of +1 and +2, respectively. The observed shift of 2.3 ± 0.2 eV is more than twice the reported value of L_3_ excitation energy shifts for nickel undergoing a change in formal oxidation state because in nickel compounds, the associated change of 3d occupation is less than unity^40^ and 3d delocalization takes place due to the nephelauxetic effect.^47^ Within the nickel halide series, we find that only in nickel fluoride [NiF]^+^ can the bonding be described as purely ionic with only one electronic configuration 3d^8^ contributing to the ground state. For the other halides in the series, we find that two configurations, 3d^9^L̲ and 3d^8^, contribute to the ground state. Since the 3d^9^L̲ configuration, including a hole at the ligand, lies lower in energy, the bonding between nickel and halide in these systems is best described in the same terms used for inverted ligand-field compounds^48,77^ or for negative charge-transfer materials.^78^ Along the halide series, as the ligand electronegativity decreases from chloride to bromide to iodide, the stabilization of the 3d^9^L̲ configuration increases, as evidenced by the increasing energy difference between the 3d^9^L̲ and 3d^8^ configurations in the initial and final states. Despite the wealth of information gathered from nickel L-edge spectroscopy, a complementary study of the ligand K-edges could confirm the existence of a ligand hole in the nickel halides exhibiting an inverted ligand field.^79^ Overall, the results presented here further illustrate that an inverted ligand field in the electronic structure of nickel compounds is quite common, and thus, anion redox chemistry often plays an important role in such materials.^80^

## Supporting Information Available

Additional experimental details, computational details, mass spectra, and X-ray absorption spectra over a larger energy range than that provided in the manuscript (PDF)
