# Supplementary material for: Abrupt Change from Ionic to Covalent Bonding in Nickel Halides Accompanied by Ligand Field Inversion
Source: Inorg Chem. 2024 Jun 10;63(25):11812–20. doi: 10.1021/acs.inorgchem.4c01547 (PMC11200264; doi:10.1021/acs.inorgchem.4c01547)
Supplement: Supplementary file 1 — ic4c01547_si_001.pdf [file ic4c01547_si_001.pdf]

Supporting Information for Abrupt change from ionic to covalent bonding in nickel halides accompanied by ligand field inversion

Max Flach,*,†,‡ Konstantin Hirsch,† Tim Gitzinger,‡ Martin Timm,† Mayara da Silva Santos,†,‡ Olesya Ablyasova,†,‡ Markus Kubin,† Bernd von Issendorff,‡ J. Tobias Lau,†,‡ and Vicente Zamudio-Bayer*,†

†*Abteilung für hochempfindliche Röntgenspektroskopie, Helmholtz-Zentrum Berlin für Materialien and Energie, Berlin*

‡*Physikalisches Institut, Albert-Ludwigs-Universität Freiburg, Freiburg*

E-mail: max.flach@helmholtz-berlin.de; vicente.zamudio-bayer@helmholtz-berlin.de

Mass Spectra

To produce nickel cations Ni^+ in the ground state configuration $3d^9$ the trapping conditions in the trap were set to relatively harsh settings to dissociate the produced $[NiAr]^+$ molecules into Ni^+ and Ar.

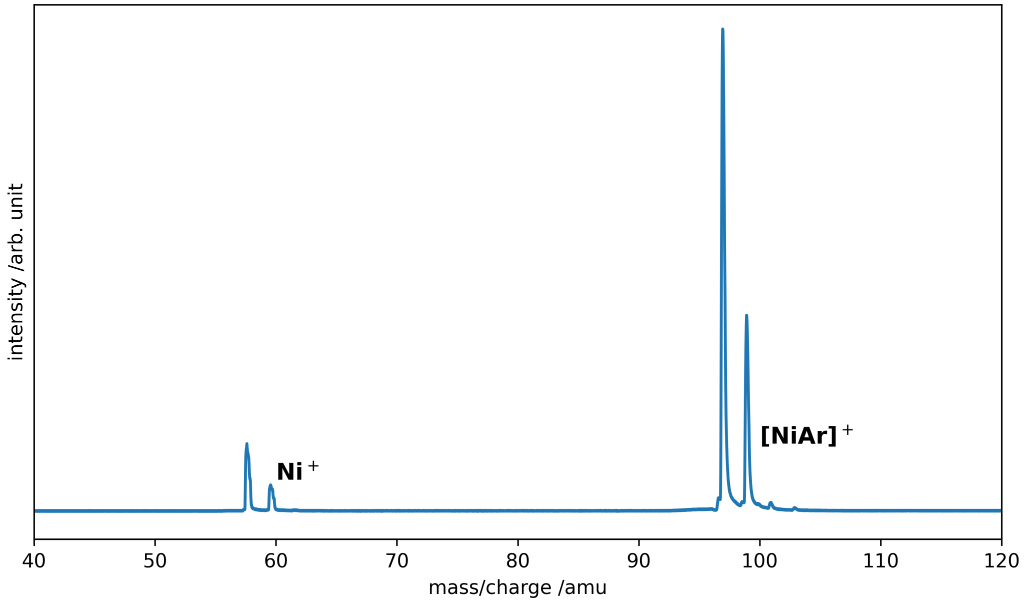

Figure 1: Mass spectrum of trapped precursor $[NiAr]^+$ under normal trapping conditions (trapping potential -1.4V)

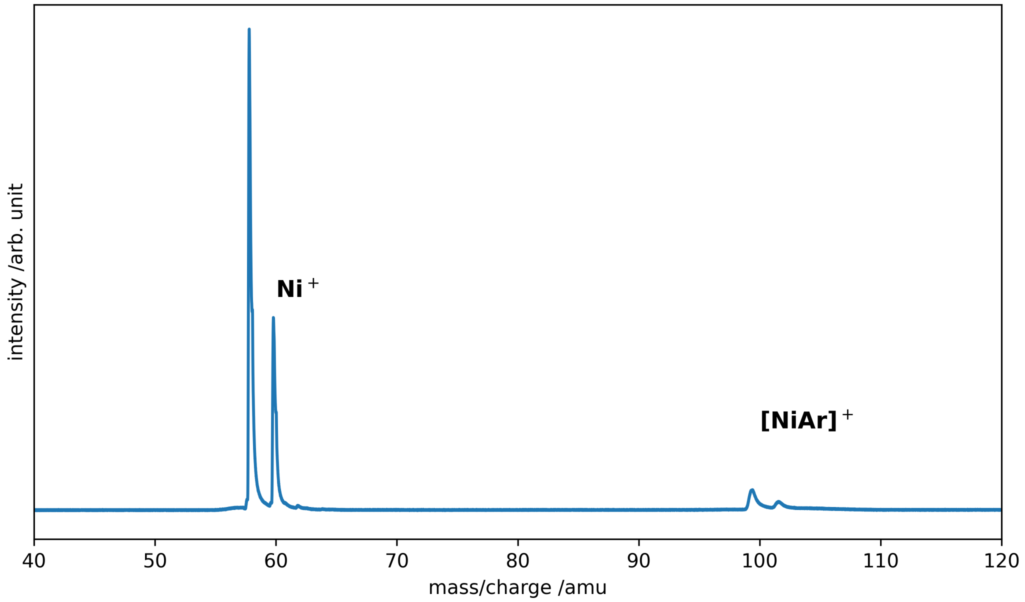

Figure 2: Mass spectrum of trapped precursor $[NiAr]^+$ under harsh trapping conditions (trapping potential -9V)

Charge Transfer Multiplet Calculations

As spectra of $[NiX]^+$ (X=Cl,Br,I) show a simple structure at the nickel L_3 resonance the determination of the best fit was done by optimizing the charge transfer calculation parameters to match the main line-satellite separation ($W_{1,2}$) (see figure 3) determined from fitting each line in the spectra (see table 1). The parameters found for the best fit are tabulated in table 2. Although different combinations Δ_{CT} and U_{pd} resulting in the same line-satellite separation value $\Delta E(W_{1,2})$ may give also good agreement with the experiment, they also lead to differing line intensities and absolute energy positions. For the extreme cases (for example for $[NiCl]^+$: Δ_{CT}=0eV with U_{pd}=1.35eV or Δ_{CT}=-1.35eV with U_{pd}=0eV) still show significant higher 3d count than the pure $3d^8$ occupation.

Because of the evident similarities between the nickel L_3-edge spectra of Ni^+ $3d^8 4s^1$ and $[NiF]^+$ it was estimated that the crystal field splitting should be small and charge transfer is negligible. Therefore the calculations where performed without charge transfer only with a crystal field. The crystal value starting from zero was increased to find the best fit considering the number of resolved multiplets, energetic separation and relative intensities.

Table 1: Separation of main line and both satellites determined by fit from experimental and calculated spectra for parameters used in calculation ,see table 2.

system	$\Delta E(W_1)$ (Exp) /eV	$\Delta E(W_2)$ (Exp) /eV	$\Delta E(W_1)$ (CTM) /eV	$\Delta E(W_2)$ (CTM) /eV
$[NiCl]^+$	2.02	4.3	2.02	4.44
$[NiBr]^+$	2.2	4.52	2.22	4.62
$[NiI]^+$	2.68	5.02	2.68	5.02

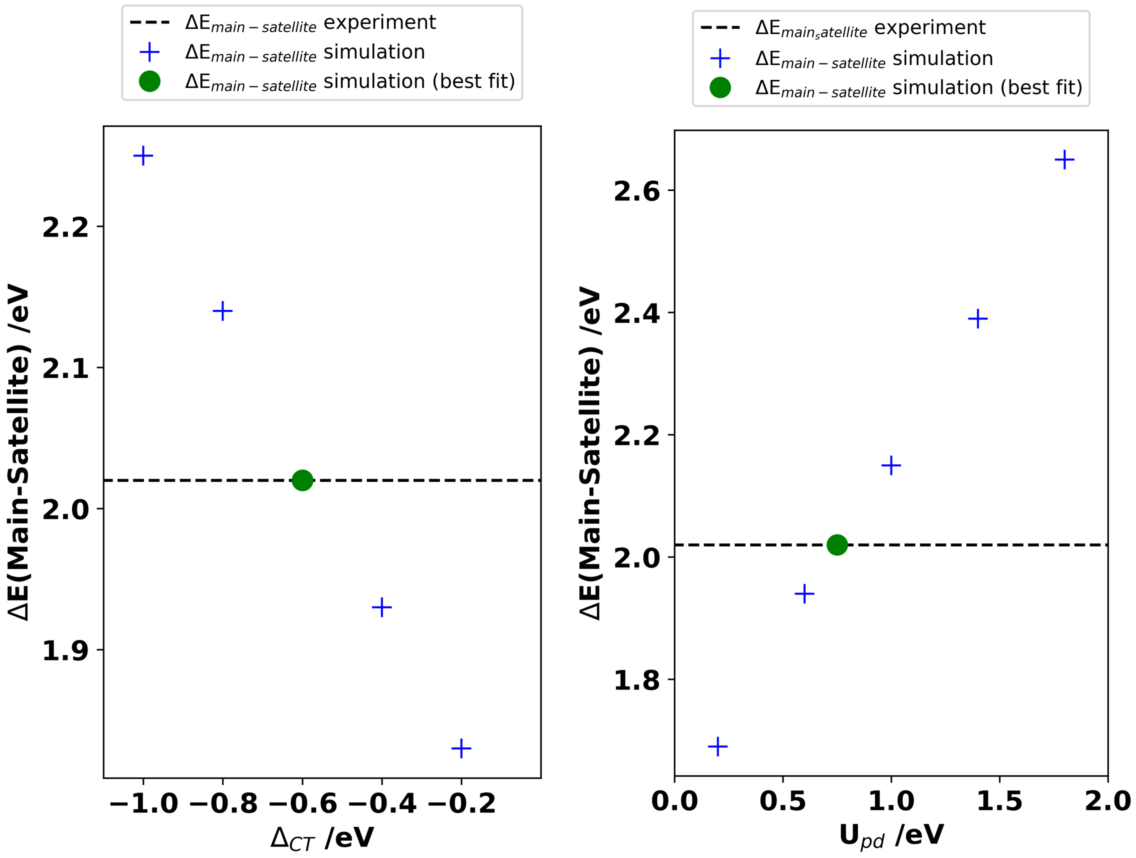

Figure 3: Splitting between the main line and first satellite in $[NiCl]^+$ for different parameters of Δ_{CT} and U_{pd} in comparison to the experimental values. On the left side the parameter U_{pd} is kept fixed at 0.75eV while on the right side the parameter Δ_{CT} is kept fixed at -0.6eV.

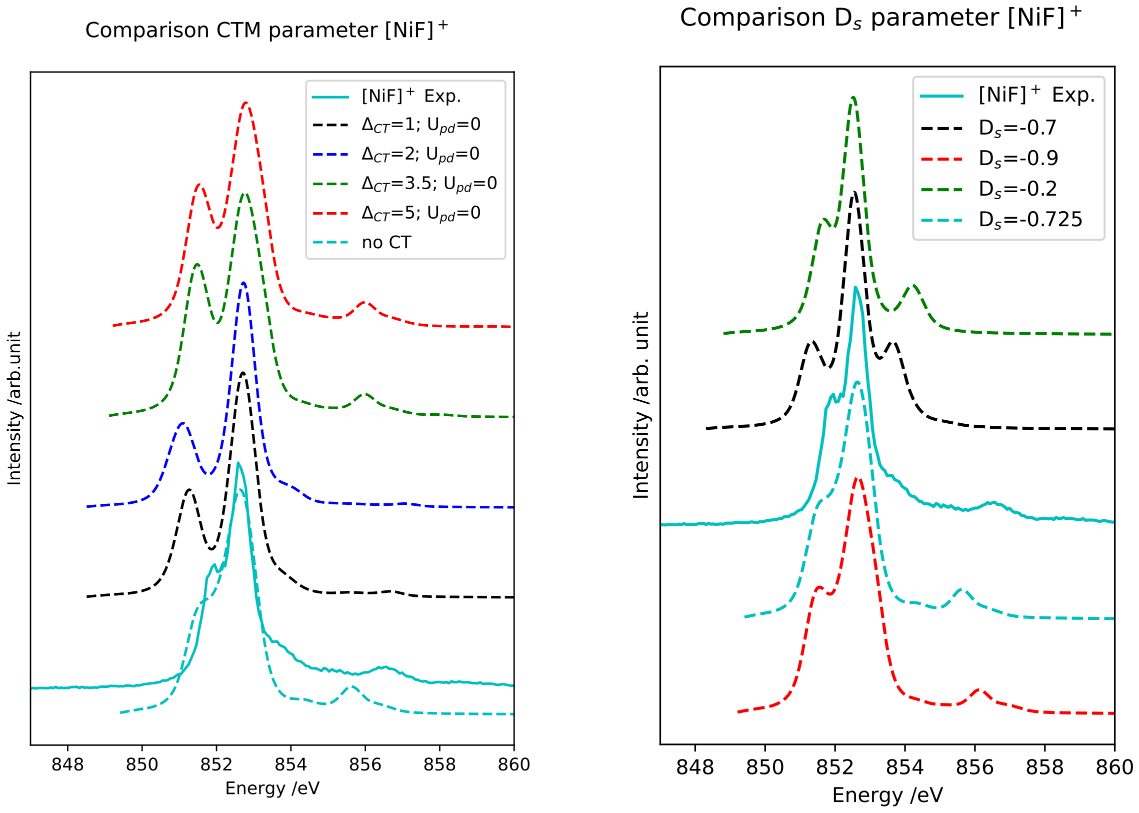

Figure 4: Left: Charge transfer multiplet calculation of $[NiF]^+$ with different Δ_{CT} values. For all calculations a crystal field value of D_s=-0.725eV is applied. It is clearly visible that the separation of the two main lines is significantly larger than the experimentally observed splitting even with large values of Δ_{CT}. Right: crystal field calculations of $[NiF]^+$ with different values of D_s without charge transfer (CT). For large values of D_s an atom-like spectral shape is reproduced. As D_s becomes more negative the spectral shape deviates more from the atomic spectrum until a abrupt change just occurs reproducing the line shape of $[NiF]^+$ reasonably well. When D_s is decreased even further the main line to satellite separation increases more towards the experimental one while the main line splitting increases as well. Hence in the calculation a D_s value was chosen as a compromise between agreement of main line splitting and main line to satellite separation.

Table 2: Values used in charge transfer multiplet calculations (CTM4XAS),[1] with a C_4 symmetry and 10Dq = 0. Note that for $[NiF]^+$ no charge transfer was used, instead there was a crystal field applied.

	Δ_{CT} /eV	U_{pd} /eV	T(b1)	T(b2)	T(a1)	T(e)	D_s /eV	D_t /eV	N_{holes} (3d)	E_{max} (CTM) /eV	shift to Exp. /eV	Gauss /eV	Lorenz /eV
$[NiF]^+$							-0.725	0	2	854.24	-1.66	0.2	0.2
$[NiCl]^+$	-0.6	0.75	0	0	2	1			1.58	852.61	-0.73	0.2	0.2
$[NiBr]^+$	-0.7	1	0	0	2	1			1.57	852.47	-0.80	0.2	0.2
$[NiI]^+$	-0.75	1.7	0	0	2	1			1.56	852.04	-0.86	0.2	0.2

Discussion of the Ground State of Cationic Nickel Halides

In the calculation of the $[NiF]^+$ the best agreement was found using only a crystal field with no charge transfer (see table 2). Because only the D_s value is non zero the ordering of the occupied nickel centered orbitals 3dδ, 3dπ, and 3dσ with energy separation of 2.175 eV and 0.725 eV.[2] This suggests a $^3\Pi_{4/2}$ ground state which is in line with literature.[3]

For the $[NiX]^+$ (X=Cl,Br,I) the three different hopping parameters ($T(b_{1,2})$, $T(a_1)$, T(e), see table 2) cause a splitting of less than 0.3 eV[1] between 3dσ and 3dπ and between 3dπ and 3dδ, that is, much smaller with respect to the spacing in $[NiF]^+$. Hence, the much closer energy spacing in the $[NiX]^+$ (X=Cl,Br,I) species makes it less obvious to derive a ground state analogue to neutral nickel halides.[4–6]

Experimental Details

Sample Preparation $[NiAr]^+$

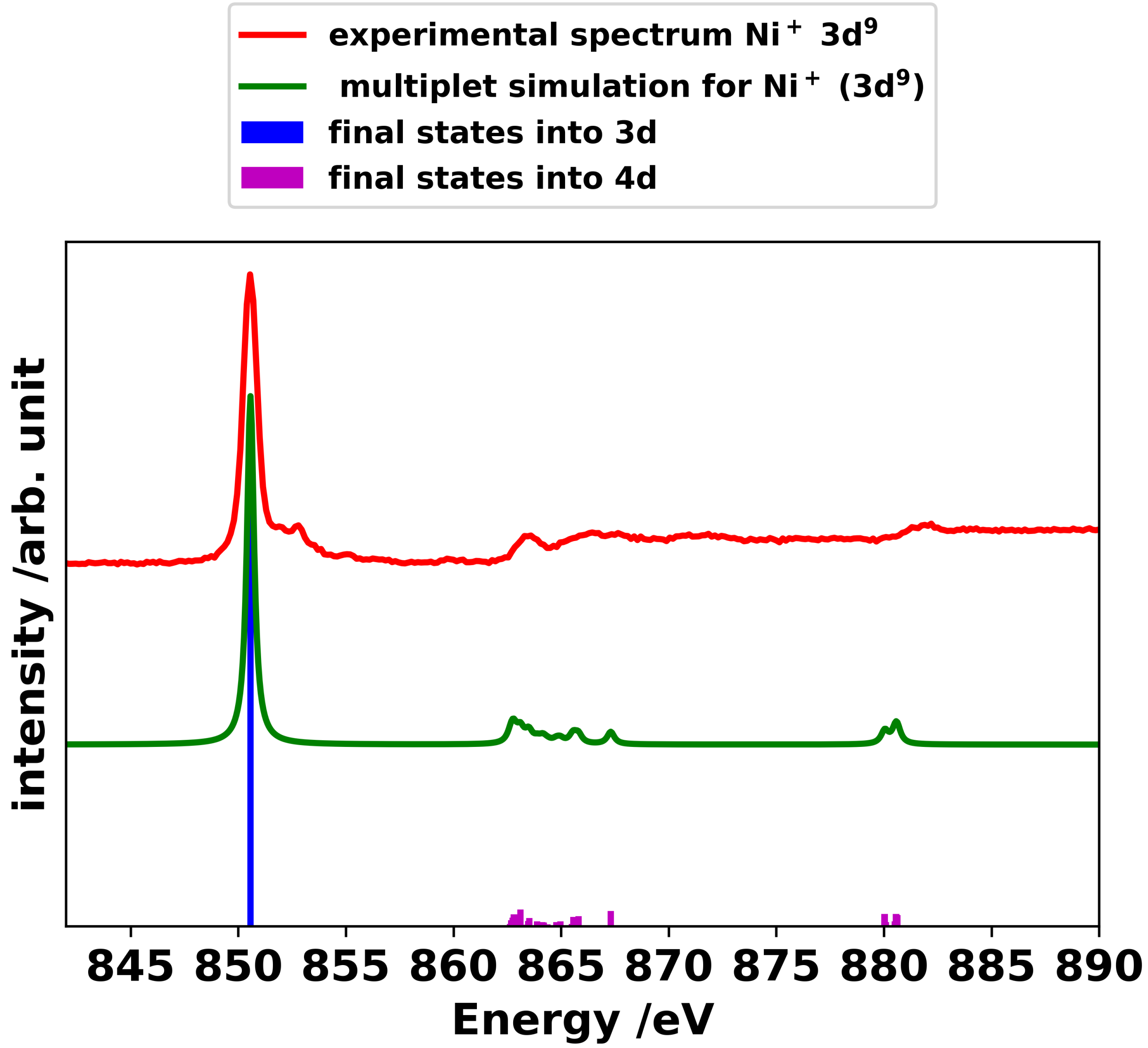

Figure 5: Experimental overview spectrum of Ni^+ $3d^9$ together with a Hartree-Fock calculation[7] (initial state $2p^6 3d^9 4d^0$; final state $2p^5 3d^1 04d^0$, $2p^5 3d^9 4d^1$). As can be seen from the calculated spectrum there is no L_2 resonance. All the intensity originates from transitions into higher orbitals and direct ionization. Because there is only one significant line at the L_3 resonance and the absence of a L_2 the hole in the initial state has to be in a δ-orbital. Hence the Ni^+ is in the ground state of $^2D_{\frac{5}{2}}$.

Experimental Parameters for Magnetron Source

Table 3: Experimental parameters used to produced ions using the magnetron sputter source

Sample	gas (Ar) /sccm	gas (N) /sccm	gas (He) /sccm	Hexgas	Source T /K
Ni^+	20	280	320	/	room temperature
$[NiAr]^+$	35	280	0	/	78.7
$[NiF]^+$	14	280	180	CH_3F	room temperature.
$[NiCl]^+$	20	282	320	CH_2Cl	room temperature
$[NiBr]^+$	20	280	390	CBr_2F_2	room temperature

Experimental Parameters for ESI Source

Table 4: Experimental parameters used to produced ions using the electro-spray ionization source.

Sample	Salt	Salt qty /mg	Solute	Solute qty /ml	c /mM	flow rate ml/h
$[NiBr]^+$	Ni(II)Br_2 (CAS: 13462-88-9)	1	H_2O	5	0.9	0.1
$[NiI]^+$	Ni(II)I_2 (CAS: 13462-90-3)	1.1	H_2O	5	0.7	0.2

X-ray absorption spectra of Ni^{+} and $[NiX]^{+}$ across nickel $L_{2,3}$

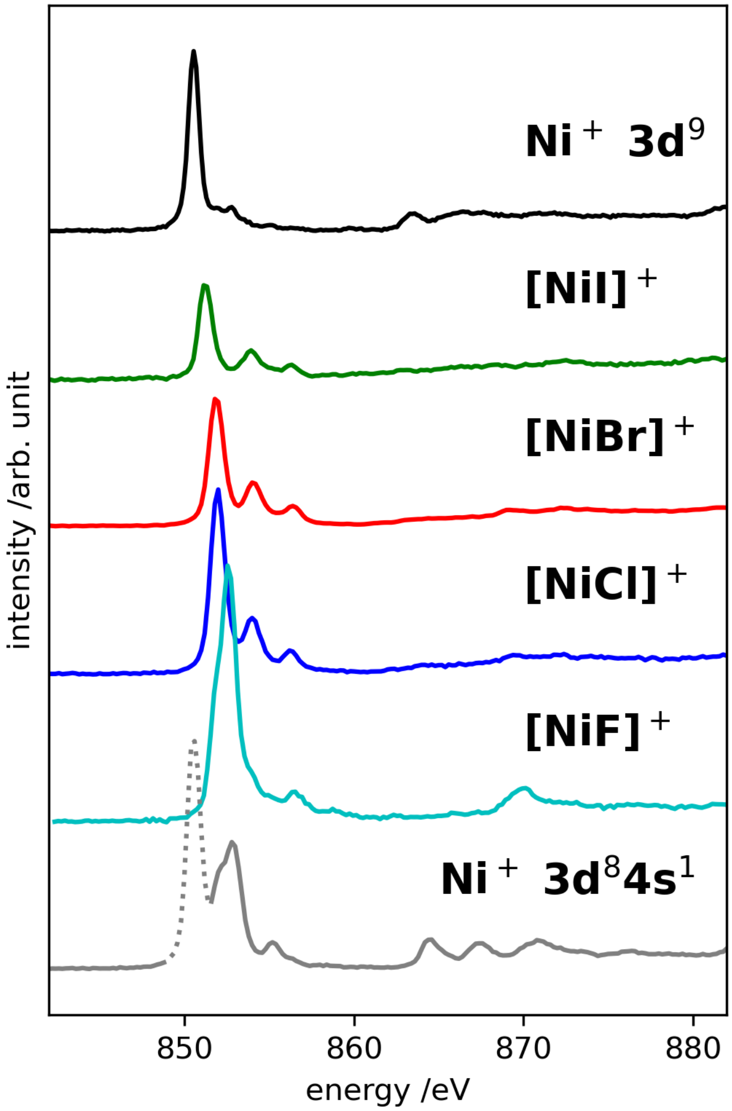

Figure 6: $[NiX]^{+}$ and Ni^{+} in $3d^{8}4s^{1}$, $3d^{9}$ configuration X-ray absorption spectra at the Ni $L_{2,3}$ edges (solid lines) normalized to 2p photo-ionization continuum. The $3d^{9}$ contamination in the Ni^{+} in $3d^{8}4s^{1}$ at the L_{3} resonance is subtracted (indicated by the dashed line).

References

(1) Stavitski, E.; de Groot, F. M. F. The CTM4XAS program for EELS and XAS spectral shape analysis of transition metal L edges. *Micron* **2010**, *41*, 687.

(2) de Groot, F.; Kotani, A. *Core Level Spectroscopy of Solids*; Advances in Condensed Matter Science 6; CRC Press, 2008.

(3) Schlangen, M.; Schwarz, H. Ligand Effects on the Mechanisms of Thermal Bond Activation in the Gas-Phase Reactions NiX+/CH4→Ni(CH3)+/HX (X=H, CH3, OH, F). Short Communication. *Helvetica Chimica Acta* **2008**, *91*, 2203.

(4) Zou, W.; Liu, W. Comprehensive theoretical studies on the low-lying electronic states of NiF, NiCl, NiBr, and NiI. *The Journal of Chemical Physics* **2006**, *124*, 154312.

(5) Tam, W. S.; Ye, J.; Cheung, A. S.-C. Near-infrared laser spectroscopy of NiI. *The Journal of Chemical Physics* **2004**, *121*, 9430.

(6) Leung, J. W.-H.; Wang, X.; Cheung, A. S.-C. Laser spectroscopy of NiBr: Ground and low-lying electronic states. *The Journal of Chemical Physics* **2002**, *117*, 3694.

(7) Gusmeroli, R.; Dallera, C. MISSING (Multiplet Inner-Shell Spectroscopy Interactive GUI). *available from ESRF* **2006**,
